# Prevalence and associated factors of self-medication in worldwide pregnant women: systematic review and meta-analysis

**DOI:** 10.1186/s12889-023-17195-1

**Published:** 2024-01-27

**Authors:** Afaf Bouqoufi, Lahlou Laila, Said Boujraf, Fatima Ait El Hadj, Rachid Razine, Redouane Abouqal, Youssef Khabbal

**Affiliations:** 1 Laboratory of Health and Science, Therapeutic Innovation, Translational Research, and Epidemiology. Faculty of Medicine and Pharmacy, Ibn Zohr University, Agadir, Morocco; 2 Clinical Neurosciences Laboratory, Faculty of Medicine, Sidi Mohamed Ben Abdellah University, Fez, Morocco; 3 Laboraoty of Epidemiology and Clinical Research, Faculty of Medicine and Pharmacy, University Mohamed V, Rabat, Morocco

**Keywords:** Meta-analysis, Pregnancy, Women’s Health, Self-medication, Pregnant women, Public Health

## Abstract

**Background:**

Self-medication during pregnancy is of great interest. The use of drugs during pregnancy requires a careful reflection on the benefits to the mother and the risks to the fetus. Selecting a drug or drugs for treating pregnant women can be difficult for clinicians owing to the various pharmacokinetic and physiological changes encountered during pregnancy. This systematic review and meta-analysis aimed to estimate the pooled prevalence of self-medication and associated factors among women during pregnancy.

**Methods:**

Searches were carried out at PubMed, Science Direct, Web of Science, and Google Scholar. The quality of the studies and the risk of bias were analyzed using the Joanna Briggs Critical Appraisal Checklist for Analytical Cross-Sectional Studies instrument. The extracted data were tabulated and analyzed qualitatively and quantitatively through meta-analysis.

**Results:**

The overall prevalence of self-medication among pregnant women was 44.50% (95% CI: 38.92–50.23). Subgroup analyses showed differences in self-medication prevalence influenced by region, county income, and study design. The Heterogeneity, assessed by the statistical test I2 varied from 96 to 99% and was statistically significant. The result of this funnel plot showed that the funnel plot was symmetry with *p*-value = 0.36, and there is no publication bias.

**Conclusion:**

The results obtained from this study showed that the prevalence of self-medication among pregnant women is relatively high. This requires effective measures and interventions to reduce self-medication.

**Trial registration:**

ID = CRD42022312333.

## Background

The World Health Organization (WHO) defines self-medication as a practice that leads individuals to treat their illnesses with authorized medicines. Self-medication can be defined as "the act of the subject, on his initiative, to consume a drug without consulting a doctor, and whether the drug is already in his possession or whether he obtains it from a pharmacy or another person [1]. It is a very prevalent practice, and the majority of medicines consumed by the population are available without a doctor's prescription [2]. The practice of self-medication is common throughout the world, both in developing and developed countries [3]. The number of people who self-medicate varies significantly by country, for example, in a study conducted in the United States about 71% of men and 82% of women had used self-medication at least once [4]. In the United Kingdom of Britain and Northern Ireland, 41.5% of people had used medicines without a prescription [5].

The use of drugs during pregnancy requires a careful reflection on the benefits to the mother and the risks to the fetus. It is a difficult medical situation for clinicians to select the drug because of the various pharmacokinetic and physiological changes encountered in pregnant women [6, 7]. Self-medication during pregnancy is of great interest; it carries serious risks of drug interactions, misdiagnosis, use of excessive doses of drugs, and prolonged use of drugs. [8, 9]. In Europe, a survey of 740 pregnant women conducted in France revealed that 41.5% of the participants in the study practiced self-medication [3]. In Africa, a systematic review conducted in Ethiopia revealed a prevalence of self-medication of 12.8% to 77.1% [7]. The studies are heterogeneous some of them report a high prevalence and others show the opposite. Also, there is one meta-analysis of the prevalence of self-medication in pregnant women that has been carried out in the world with a small number of included studies [10]. The real objective of this study was to update the published data. The goal of this study was to estimate the pooled prevalence of self-medication in worldwide pregnant women through a systematic review and a meta-analysis of published studies on self-medication during pregnancy.

## Methods

The steps of this systematic review complied with the recommendations in the PRISMA (Preferred Reporting for Systematic Reviews and Meta-Analysis) grid. A systematic review protocol was registered by PROSPERO.55 with ID = CRD42022312333. In this study, the focused research question was: “What is the prevalence of self-medication in pregnant women in the world?” [10].

### Eligibility criteria

We included all published articles between January 2011 and December 2021. We also included all original studies published in English and French and human studies of pregnant or postnatal women. Studies or scientific medical literature were also included if they described the prevalence of self-medication. Only observational studies of cross-sectional type were included, as well as cohort studies if they mentioned the prevalence of self-medication in pregnant women.

We excluded non-English studies (Persian and Spanish) if they didn’t contain the requested data. We also excluded unpublished reports, pilot studies, conference abstracts, opinion articles, editorial reports, seminal work, systematic reviews, and animal research.

### Information sources and search strategy

To collect the maximum of data, we performed an exhaustive bibliographic search in PubMed, Science Direct, Google Scholar, and Web of Science databases started in December 2021. It was performed to identify all relevant studies available from January 2011 to December 2021. The search was performed using Boolean operators AND or OR that narrowed the search and also using a combination of terms and medical subject heading (MeSH). For example, the search strategy in PubMed was as follows: (((self care[MeSH Terms])) OR (self-medication [MeSH Terms])) AND (pregnan*[MeSH Terms]).

All stages of the search were performed by 2 independent researchers (B.A) and (L.L). Any disagreements were resolved by a third researcher (K.Y).

### Selection process

A screening strategy was implemented to identify all relevant studies. We used Covidence, software that was developed by an Australian in 2015, it’s the primary screening and data extraction tool for Cochrane authors conducting standard intervention reviews. Covidence is designed to perform the following functions to make review production more efficient. Initially, duplicates were eliminated by the software. Then secondly, the initial selection was made according to the abstract and title. This was followed by reading the full text to identify eligible studies. In case of disagreement, a third reviewer (K.Y) was asked to make a clean agreement. Finally, the articles included in this review were downloaded and the references of each article were manually searched to determine whether other studies met the criteria.

### Study risk of bias assessment

The quality assessment of eligible studies was reviewed by two independent reviewers (B.A) and (L.L). This process was conducted using a recent version of the Joanna Briggs Institute's critical appraisal tools (Checklist for Analytical Cross-Sectional Studies and Cohort studies) [11, 12]. Joanna Briggs Institute (JBI) is an international research organization based in the Faculty of Medical and Health Sciences at the University of Adelaide in South Australia. [13]. The purpose of this process is to assess the methodological quality and risk of bias in included studies. This checklist is divided into eight items for cross-sectional studies and ten items for cohort studies. Each item is scored with one point. A study was considered low quality if it had 0–4 points, moderate quality if it had 5–6 points, and high quality if it had 7–8 points.

A third reviewer (Y.K.) was consulted if a consensus could not be reached. When information was missing from the studies, we tried to contact the authors by e-mail. All cross-sectional and cohort studies were included, regardless of their quality score. Articles with missing data were included as long as they reported the prevalence of self-medication.

The results of the quality assessment of eligible studies are presented in Fig. 2.

### Effect measures

What is the prevalence of self-medication? The prevalence is the proportion of pregnant women who have self-medicated or taken drugs without a doctor’s prescription during their pregnancy period.

### Data extraction

Data covering author, country, year of publication, sample size, age of participants, and prevalence of self-medication were extracted and collated in an Excel table. We also extracted the associated factors of self-medication including the reason for use, source of information, illness of drug used, type of drugs used, and finally sociodemographic characteristics of pregnant women. Any disagreements were resolved by consensus with a third reviewer (K.Y). For non-English studies, we extracted data from the abstracts available in English.

### Statistical analysis

To calculate the pooled prevalence, we used a random-effects model, calculating effect sizes with a confidence interval (CI) using Wilson-Score, and Clopper Pearson methods [14] To estimate the true treatment effects that can be expected we calculated the prediction interval using the method that are described by Barker et al. (2021) [15]. The Cochrane criteria such as I2 < 40%—insignificant, 40- < 75% is moderate while 75% + is considerable [16].

To investigate the effect of variables on heterogeneity, we performed a subgroup analysis by ranking eligible studies by income (based on the country in which the study was conducted). This was done by consulting the World Bank website [17], study region, sample size, and study quality. The R software was used for the statistical analysis. In the R4.1.2 version, we used the Meta and Metafor packages for the metanalysis.

### Sensitivity analysis

To identify and reduce the source of heterogeneity, we conducted sensitivity analysis based on studies with small sample sizes (≥ 500 participants), and studies with a high risk of bias in any methodological domain (studies that have a low-quality score).

## Results

### Study selection

**Fig. 1:**
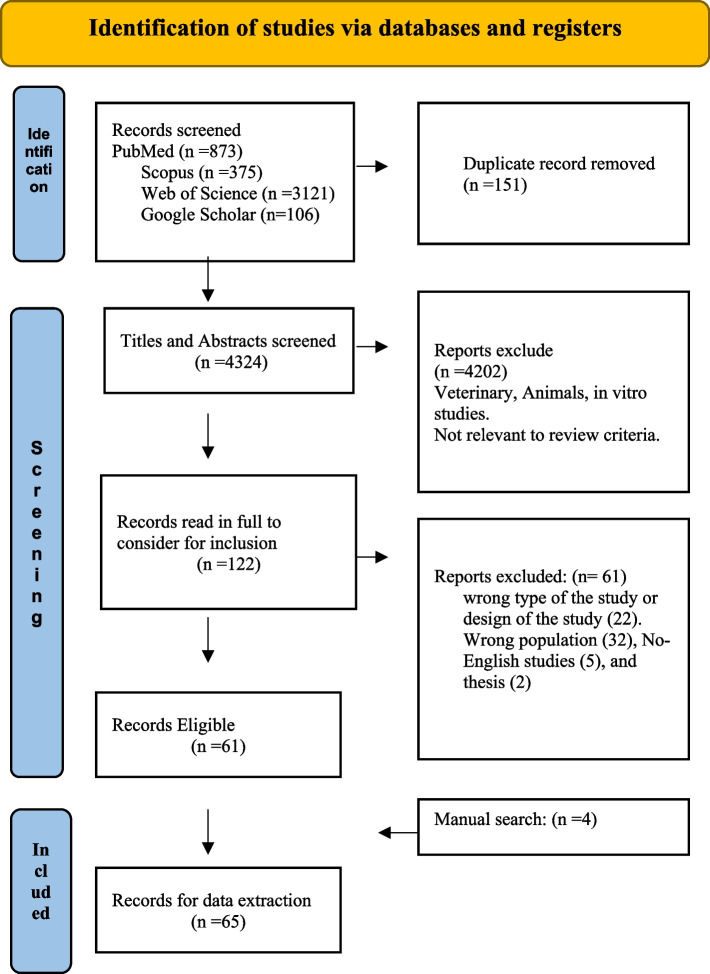
Systematic review flowchart

The flow chart of the studies included in this systematic review is shown in Fig. 1. The search generated 4475 articles, 151 of which were duplicates and subsequently deleted. We examined the titles and abstracts of 4324 articles. In addition, 4202 were excluded as ineligible based on the inclusion and exclusion criteria of the analysis. Then we examined the full text of the remaining 122 articles for eligibility. We, therefore, excluded 61 studies the reason (of the wrong target population (32), inappropriate study design (22), non-English articles (5), and thesis (2)), therefore 59 studies were eligible and they proceeded to the data extraction after adding four articles from the manual search. Finally, 65 studies were eligible and included in the present systematic review.

### Characteristics of included studies

The total sample size analyzed included 42,615 pregnant women. The studies were published between 2011 and 2021. All included studies were cross-sectional observational studies except three studies were Cohort [18–20]. The oldest study in terms of publication year (2011) is [21]. The most recent studies published in 2021 are (Table 1).

**Table 1:** Main characteristics of the included studies

References	Country	Year of publication	Study design	Sample size	Prevalence	Age
Atif [22]	Pakistan	2018	cross sectional	351	37.9	26.28 ± 10.4
Abasiubong [23]	Nigeria	2012	cross sectional	518	72.40	< 20 8,7%
Abduelkarem [24]	United Arab Emirates	2017	Cross sectional	140	40.0	15–20 (2.1%) 21–25 (25%)
Abeje [25]	Ethiopia	2015	Cross sectional	510	2510	26.5 (± 6.0)
Adanikin [26]	Nigeria	2016	Cross sectional	346	31.50	< 20 (1.4)
Afshary [27]	Iran	2015	cross-sectional	801	30.60	Less than 25 37,8%
Seid Mussa Ahmed [28]	Ethiopia	2020	cross-sectional	1117	27	18–45 years old, with a median age 25 years
Ake [29]	Nigeria	2021	cross-sectional	359	40.40	26.92 years (SD = + 5.057
Alonso-Castro [30]	Mexico	2018	A cross-sectional	1798	21.90	< 25 37,6%
Alsous [31]	Jordan	2020	cross-sectional	1313	33.10	31.11 ± 5.71
Araujo [32]	Brazil	2013	cross sectional	78	28.2	NR
Atmadani [33]	Indonesia	2020	cross-sectional	333	11.7	16–27 41%
Befekadu [34]	Ethiopia	2014	cross-sectional	315	20.10	Mean = 22.58 SD = 4.298
Beza [35]	Ethiopia	2018	cross-sectional	617	26.6	18–24 (31.4)
BiBintsene [36]	Congo	2018	cross-sectional	350	46.24	67 + 6,3
Bobga [37]	Cameron	2021	cross-sectional	500	67.40	15—20 23.0%
Bohio [38]	Pakistan	2016	cross-sectional	351	36.5	26.19 ± 4.82
Malihe Botyar [39]	Iran	2018	cross-sectional	210	34.8	> 20 0%
Bulabula [40]	South Africa	2019	cross-sectional	301	16.60	29 (6.1) years
Cabut [41]	France	2017	A cross-sectional	68	72	[30.2 ± 3.9]
Navaro [42]	Italy	2018	cross-sectional	503	59.6	NR
Ebrahimi [43]	Iran	2017	cross-sectional	384	43.5	26.33 ± 4.60
Gbagbo [44]	Ghana	2020	cross-sectional	136	69	29 ± 5
Grover [45]	USA	2019	cross-sectional	138	53%	Nr
Haque [46]	Bangladesh	2016	cross-sectional	650	12.2	15–20 35.4% 21–25 30.4%,26–30 24.1%, 31–35 7.6%,36–40 2.5%, > 40 0%
Barhamji [47]	France	2014	cross sectional	330	46	29,7 ± 5,3 ans
Ibrahim [48]	United Arab Emirates	2021	cross-sectional	434	41	< 20 2%,20–29 31.3%, 30–39 37.6%,40–60 29.1%
Jambo [49]	Ethiopia	2018	cross-sectional	244	69.4	25 ± 4.68
Kabamba [50]	Congo	2014	cross sectional	145	75	16–20 15,1%, 21–30 57,9%
Karami Matin [51]	Iran	2016	cross-sectional	308	29.3	19–43 29.16 ± 6.44
Leke [52]	Cameroon	2018	cross-sectional	795	73.2	13–17 5.2% 18–25 47.8%, 285 75.0%,26–35 42.1%
Liao [53]	China	2015	cross-sectional	422	2.6	20- 42,29.57 ± 3.966
Lutz [19]	Brazil	2015	Cohort	4270	27.7	NR
Marwa [54]	Tanzania	2018	cross-sectional	372	46.24	18–27 47.06% 28–37 45.32% 38–47 45.45% > 47 0%
Mbarambara [55]	Congo	2016	A cross-sectional	920	59.9	15–19 28.0%,20–24 38.6%, 25–29 19.3% 30–35 11.0%, > 35 3.0%
Mehmood [56]	Pakistane	2018	A cross-sectional	351	63.5	18–45 26.28 ± 10.42
Niriayo [57]	Ethiopia	2021	A cross-sectional	250	40.8	< 18 0.8% 19–25 46%,26–30 33%, 31–35 13.6%, > 35 6.4%
Odalovic [21]	Serbia	2012	cohort	218	34.7	NR
Ojo [58]	Nigeria	2020	cross-sectional	343	48.9	15–20 3.2% 21–26 29.7%,27–32 43.4%, 33 and above 23.6%
Pakseresht [59]	Iran	2020	cross-sectional	598	8.7	16–25 24.4%, 26–35 59.7%, 36–45 15.4%, > 46 0.5%
Pereira [60]	Brazil	2021	cross-sectional study	297	36.0	< 30 43.9%,30–39 52%, > 40 7.4%
Pisa [20]	Italy	2015	Cohort	767	39.8	NR
Rafiee [61]	Iran	2018	cross-sectional	400	25.8	< 25 20.5% 25–30 30.4% > 30 72.6%
Raheel [62]	Saudi Arabia	2017	cross-sectional	354	32	< 20 2.5%,20–30 67%, 31–41 25.5%, > 40 5%
Sah [2]	Nepal	2020	this cross-sectional	225	41.3	24–29 41.3%
Sema [63]	Ethiopia	2020	this cross-sectional	400	44.8	28–37 27.83 ± 4.27
Adama [64]	Ghana	2021	cross-sectional	367	74.1	18–24 19,6%, 25–34 68.9%, 35–45 11.5
Tefera [65]	Ethiopia	2020	cross-sectional	423	18.2	27.22 ± 5.5
Tuha A [66]	Ethiopia	2020	cross-sectional	223	26.9	15–19 26% 20–24 10.8%, 25–29 31.8%, 30–34 16.6% 35–39 10.8% 40–49 4%
Verstappen [67]	Pays bas	2013	cross-sectional	1246	12.5	18–35 23%, > 35 15.1%
Wakjira [68]	Ethiopia	2019	cross-sectional	195	21.5	30.4 + 3.45
Yusuff [69]	Nigeria	2011	cross-sectional	1594	63.8	NR
Zaki [70]	Saudi Arabia	2013	cross-sectional	760	40	20–30 34.2%, 30–40 44.7%, 40–50 21.1%
Zewdie [71]	ethiopia	2018	cross-sectional	323	15.5	15–24 29.1%, 25–34 63.8%
Andy Emmanuel [72]	Nigeria	2014	cross-sectional	120	85	15–20 16.6%, 21–25 37.5%,26–30 18.3%, 31–35 15%, 36–40 9.16%, > 40 3.33%
M. Sarani, S. Saravani [73]	Iran	2017	cross-sectional	350	46.6	31.4 ± 7.3
M. J. Twigg1 [74]	UK	2016	cross-sectional	856	76.4	30.5 (5.2)
D. Courrier [6]	france	2015	cross-sectional	740	41,5	30,4 (± 4,6)
Lupattelli [75]	Ausralia	2014	cross-sectional	217	77.4	NR
	Italy			926	44	
	austria			82	62.2	
	Switzerland			618	72.5	
	france			374	71.9	
	Pays bas			81	85.2	
	UK			1120	82.1	
	Norway			1288	70.4	
	Sweden			877	79.3	
	Finland			574	84.8	
	Iceland			71	81.7	
	Russia			1008	66.4	
	Poland			679	57.1	
	Croatia			286	39.7	
	Serbia			220	45.5	
	Slovenia			149	52.3	
	USA			297	60.6	
	Canada			236	68.6	
	South America			346	45.1	
Lunardi-Maia [76]	Brazil	2014	cross-sectional	212	46.7	NR
Maslakpak [77]	Iran	2013	cross-sectional	116	27.6	NR
Shah [78]	Pakistan	2021	cross-sectional	205	79	NR
Rahbar [79]	Iran	2017	cross-sectional	226	74.3	NR
Baghianimoghadam [80]	Iran	2013	cross-sectional	180	35	NR
Miní E [81]	Peru	2012	cross-sectional	400	10.5	NR

One study used the largest sample size of 9,459 participants. This was a multinational study conducted in Europe (western, northern, and eastern), North and South America, and Australia [75]. The study carried out in France recruited only 68 participants [41]. The ages of the participants ranged from 15 to 60 years. Regarding the region where the studies were conducted, 26 (31.32%) studies were conducted in Africa, 25 (30.12%) in Asia 21 (25.30%) in Europe, 10 (12.04%) in America, 1 (1.20%) in Australia.

Of most of the studies 29 (34.93%) were conducted in high-income countries, 28 (33.73%) in low-middle-income countries, 14 (16.86%) in low-income countries, and 12 (14%0.45) in upper-middle-income countries. Regarding the quality of the studies, 42 (50.60%) articles were high quality (score of 7–8), 33 (39.75%) medium quality (6—5), and 8 studies (9.63%) low quality (4—0).

### Risk of bias in studies

**Fig. 2:**
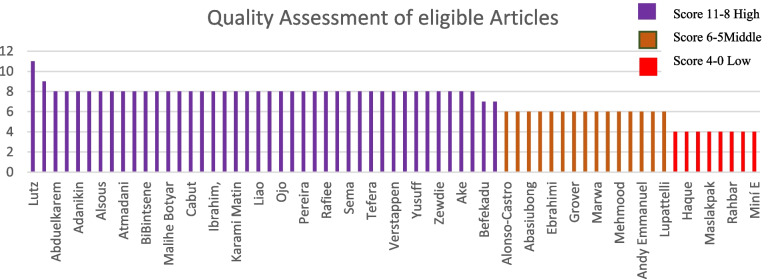
Result of quality appraisal using JBI appraisal tool

The findings of the quality appraisal of eligible studies were reported in Fig. 2. The tool is used to indicate the methodological quality and appropriateness of the observational studies, including cross-sectional and cohort studies that were reviewed in this study. We determined the score by counting the asterisks (*) that we gave to each answer to the items in the grids, where a high score (11–8) indicates a higher quality of a study, a Middle score (6–5) indicates middle quality and Low Score (4–0) indicates low quality. The sex of the sixty-five studies was evaluated by the abstract since these articles are in Spanish and Persian language and we have no response from their authors to retrieve the full text. Two reviewers completed this process, and where there were discrepancies, a team of reviewers intervened to resolve them.

### Meta-analysis of the prevalence of self-medication in pregnant women

**Fig. 3:**
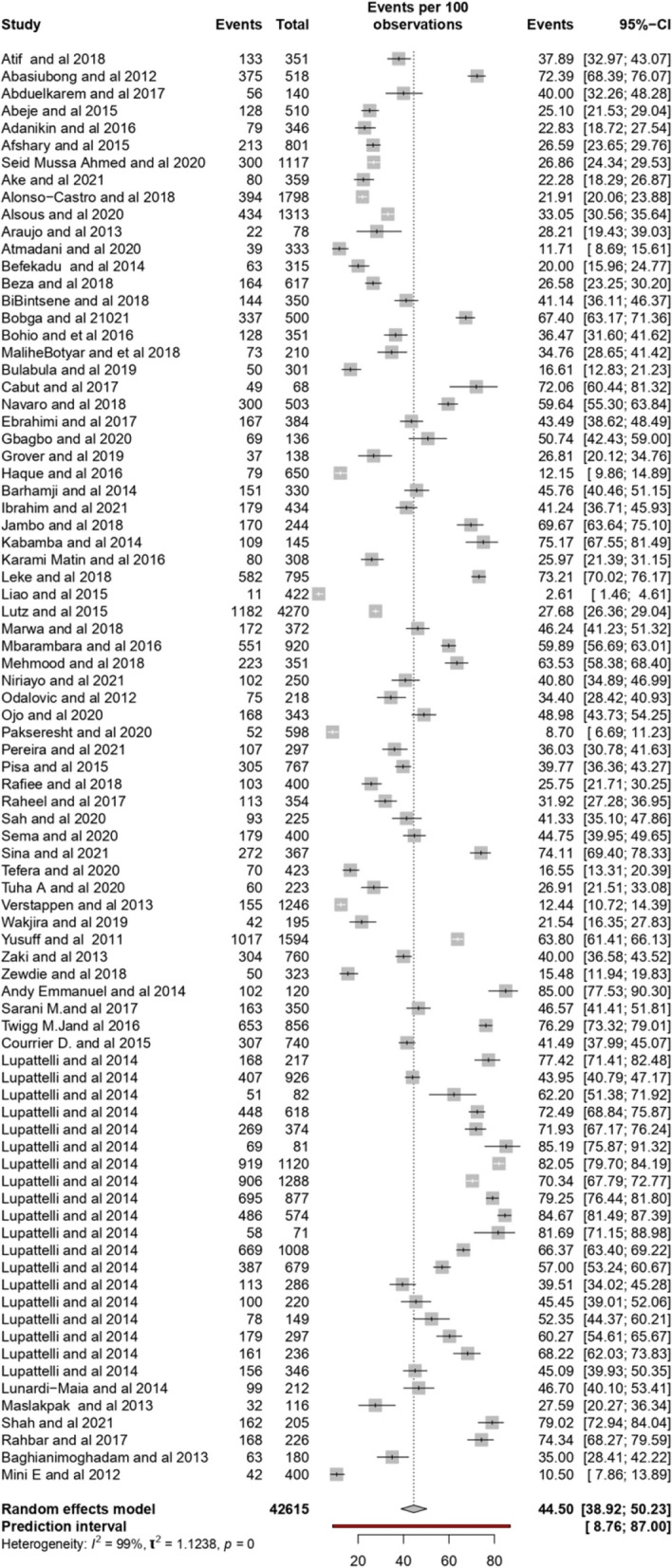
Forest Plot of the prevalence of self-medication among pregnant women

The overall prevalence of self-medication among pregnant women was 44.50% (95% CI: 38.92–50.23) with a prediction interval (of 8.76–87.00) Overall the prevalence ranged from 2.61% to 85%, as shown in the forest plot (Fig. 3). The I2 test revealed a high statistically significant heterogeneity of 99%.

**Fig. 4:**
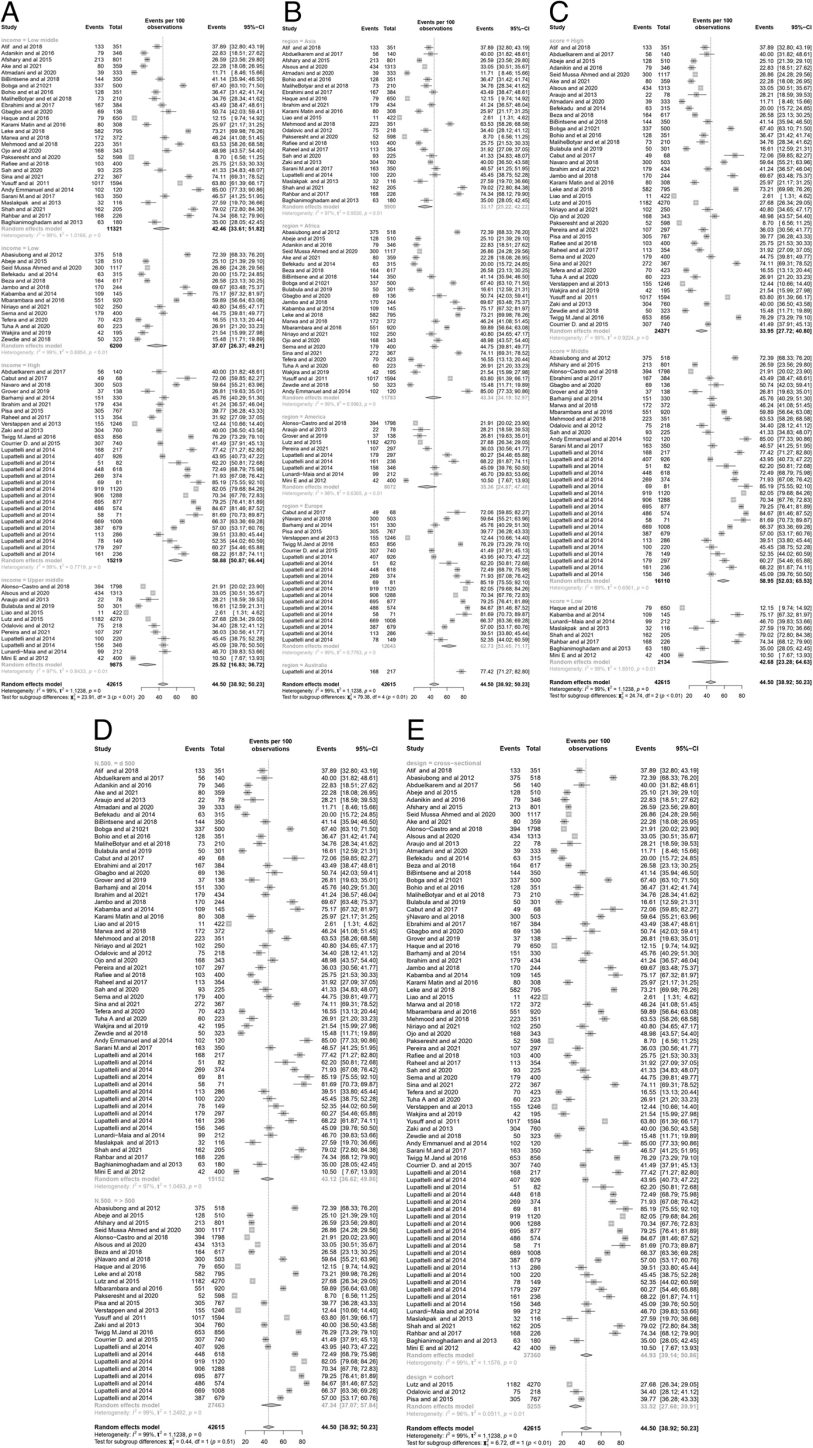
Forest Plot of subgroups analysis based on Income Level (**A**), Region (**B**), Quality of studies (**C**), Sample size (**D**), and Study Design (**E**)

Results of the subgroup analysis are shown in Fig. 4 (A, B, C, D, E). Analysis was based on income level, geographic region, quality of publication study, sample size, and study design. Heterogeneity, assessed by the statistical test I2 was considerable, statistically significant for all subgroup analyses, and ranging from 96 to 99%. By region, the highest prevalence of self-medication was 77.42% (95% CI: 77.27–82.80%) in Australia and the lowest was 33.17% (95% CI: 25.22- 42.22%) in Asia (Fig. 4B). Stratifying by a score of the quality assessment of the studies, the prevalence of self-medication was 33.95% (95% CI: 27.72–40.80%) in studies with high scores. 58.95% (95% CI: 52.03–65.53%), and 42.68% (95% CI: 23.28–64.63%), in middle and low quality respectively.

For the cohort studies, the prevalence was lower 33.52% (95% CI: 27.68- 39.91%), and 44.93%(95% CI: 39.14- 50.86%) for the cross-sectional studies.

### Sensitivity analysis

**Fig. 5:**
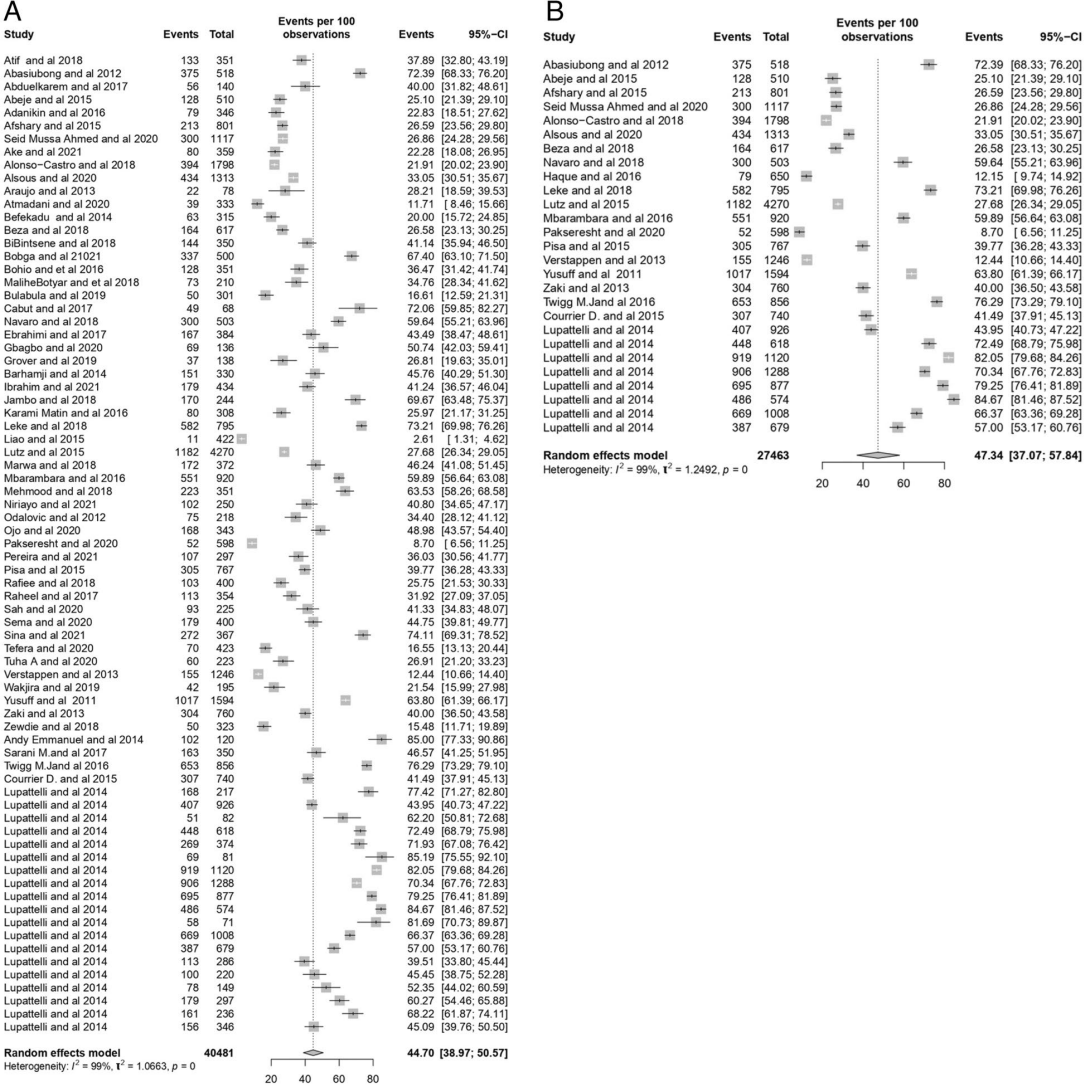
Sensitivity analysis of the stability of the results of panel **A** studies with a high risk of bias and panel **B** studies with small sample size

Due to the high heterogeneity of the results, sensitivity analysis was done after excluding studies with a high risk of bias (Fig. 5A) and studies with small sample sizes (Fig. 5B). The sensitivity analysis showed the stability of the results. The overall prevalence of self-medication based on the random effect model was determined to be 43.70% and 47.34% for the studies with a low score and small sample size, respectively. The results showed that the prevalence of self-medication did not generally change confirming the robustness and reliability of our findings.

### The reason and motivation for self-medication practice

The duration for making decisions regarding self-medication varied significantly, depending on factors such as women's health requirements, the availability of information, and personal preferences. Several studies found that women's individual choices and decisions were influenced by their personal preferences. There are many factors responsible for self–medication practice among pregnant women. The majority of the respondents pointed out that drugs are easily available in drug stores or pharmacies or by the availability of old prescription that helps themes to purchase drugs. Others think that they have better knowledge about the disease and the treatment, they know that the medication is safe during pregnancy. This report is logical when the respondents see that those illnesses as minor diseases will definitely not worry about visiting the health facility for professional care and will therefore prefer to buy non-prescribed drugs from the patent medicine shop for treatment. Especially when costly medical are expensive and the a lack of healthcare insurance, women consider self-medication as a cheaper practice. Others reported that the previous medication good experiences are one of the reasons for self-medication.

### Self-medication for health problem treatments

Most pregnant women practice self-medication on the given health condition/diagnosis and accompanying treatment plans, and the desire to get over the pain, the complication, or if infertility persisted over time. Pregnant women practice self-medication to alleviate pregnancy-associated symptoms. Antiemetics, antiacids, and antipain are the most frequently drugs used to treat gastrointestinal disorders such as nausea, vomiting, abdominal pain, bloating, flatulence, and stomach aches followed by antibiotics and analgesics to treat cold and flu symptoms. Other women treat anemia and weakness with vitamins, calcium supplements, antianemia, folic acid, and iron. Some African studies reported that pregnant women self-medicate with Antimalaria to treat Malaria (Table 2). Whereas others reported self-medication practices with anti-inflammatory antirheumatic, anthelminthic, and antiepileptics. Other uses were specifically for the stimulation of labor or facilitation of labor and delivery. Finally, skin problems, sleep disorders, and weight loss are the fewer reasons that we have marked among some users. Illness drugs used for and family therapeutics of drugs are reported in Table 2.

**Table 2:** General description of Associated Factors of self-medication among worldwide pregnant women

References	Year of publication	Country	Mean age ( Years)	Educational level( years of education)	Employement status	Residence	Scoicio Economics	Gravidity	Type of drugs	llness OTC used for	Source of recommendation	Reason of use	Religin	Ethnicity
Atif	2018	Pakistan	18–26 13.6% 27–3554.5% > 35 31.8%	Illiterate 43.3% Primary 36.2% Secondary / Higher 20.5%	Housewife 95.2% Working 4.8%	Urban 34.5% Rural 43% Semi—urban 22.5%	Low 57.3% Middle 41.6% Upper 1.1%	Primigravida 21.9% Multigravida 67.2% Grand multigravida 10.8%	Acetaminophen + Aspirin 43.6% Acetaminophen + ibuprofen 18% Multiple drugs 12% / Ibuprofen15% / Multivitamins/Iron 3.8% Acetaminophen + Antacids 2.3% Aspirin 1.5% / Antibiotics 1.5% / Castor oil 0.8% / Unknown drugs 0.8%	Headache 60.2%Multiple complains 19.5% Headache + Backache 10.5% Fever 2.3% Headache + heartburn 2.3% Weakness 2.3% Constipation 0.8% Cough 0.8% Common cold 0.8% Suicide attempt 0.8%	Yourself 73.7% Yourself and husband 13.5% Pharmacist / drug store 11.3% Neighbors / Family members 1.5%	Better Disease knowledge 11.3% Attending health care is costly 31.6%. Easily Available 27.6% / timw saving 28.6% / Suicide Attempt 0.8%	NR	NR
Abasiubong	2012	Nigeria	< 20 8.7% 20–29 44.6% 30–39 41.3% > 40 5.4%	No formal education 9.9% Primary school 19.5% Secondary school 43.8% Post-secondary school 26.8%	Full-time h/wife 14% Farming 10.4% Business 54.4% Civil servant 21.2%	NR	NR	NR	Sedatives (Lexotan, Valium, Phenobarb) 2.9% Analgesics (Pain relievers or killers) (30.3% Antibiotics (Septrin,Tetracycline, Ampicllin) 26.% Mixture native herbs and other drugs 9.1% Alcohol (P/wine, local liquor, beer, gin) 2.5%	NR	NR	NR	NR	NR
Abduelkarem	2017	United ArabEmirates	15–20 (2.1%) 21–25 (25%) 26–30 (36.4%) 31–35 (20.7%) 36–40 (10%) 41–55 (5.7%)	Primary/secondary school (7.1%) High school (25%) University or college (63.6%) Other (4.3%)	Student (6.4%) Housewife (65.7%) Healthcare personnel (physician, nurse, or pharmacist) (8.6%) Employed in the non-healthcare sector (16.4%) Other (2.9%)	Local (30%) Arab (nonlocal) (57.1%) Non-Arab (nonlocal) (12.9%	NR	NR	Folic acid (36.2%) Calcium (28.6%) Iron (35.1%) Others (8.4) Magnesium (1.4%) Multivitamins (2.1%) Panadol (55.1%) Ibuprofen (10.3%) Panadol All in One (Cold and Flu) (19.2%) Prospan or any cough syrup (14.1%)	Cold and Flu	NR	NR	NR	NR
Abeje	2015	Ethiopia	15–19 5 20–34 97 35–42 26	Has not completed primary school(46%). Completed at-least primary school(54%)	None: 20%. Peasant agriculture, Small business, Other (80%)	Urban 64. rural: 64	100–800: 42 801–1000: 33 > 1000: 53	1:(46). > 1: (62)	NR	NR	NR	NR	Christian 98% Others 2%	NR
Adanikin	2016	Nigeria	20 5 (1.4) 21–30 172 (49.7) 31–40 167 (48.3) 41 2 (0.6)	Primary (12.7%) Secondary (30.9%) Tertiary (56.45%)	NR	NR	NR	NR	NR	Asthma (0.9) Anorexia/Vomiting (8.7) Breathlessness (0.9) Cold/Flu (4.6) Cough (8.1) Dermatological disorders (0.9) Diabetes (0.6) Diarrhoea (1.7) Epilepsy 0 (0) Fatigue/Malaise (4.3) Haemorrhoids (2.3) Headache (16.2) Heartburn (3.5) Hypertension (2.3) Imminent miscarriage (1.2) Insomnia (1.7) Malaria fever (51.7) Nervousness (0.6) Constipation (0.6) Oedema 66 (19.1) Pains (3.8) Urinary tract infections (3.2) Vaginal infections (14.2) Other infections (1.2)	NR	NR	NR	NR
Afshary	2015	Iran	Less than 25 Years 42% 25–30 years 38% More Than 30 years 20% Education Level Lower Than 30.2%	Diploma 32.24% Being Educated in University 37.56%	Housekeeper 71% Employed 29%	NR	NR	0 137.9) 1–2 186 (53.8) 3–4 27 (7.8) 5 2 (0.6)	NR	Digestive disorders (38.80%), disorders related to pregnancy (30.52%), genitourinary disorders (25.26%) respiratory problems (8.42 5), antibiotics (44.8) anemia (14.2%), skin problems (13.2%) and cold (8.1%) hypoglycemia (17.8%), treatment of anemia (5.1%) and treatment of renal disorder (5.1%)	NR	Convenient Access 5.2% Mild Disease 19.8% Pre-experience of the disease 49% Not taking serious the disease 4.2% Costly medical expenses 35.4% Lack of healthcare insurance 2.1% Lack of time 2.1% The drugat home 2.1% Experiencing good resultfromself-medication 15.6% Lackof faith indoctors practice 3.1% Harmlessherbs and medicinalplants 3.1% Inexpensive herbs and medicinal plants 0% herbs and medicinal plants 1% Using before pregnancy 1% Other 13.5%	NR	NR
Seid Mussa Ahmed	2020	Ethiopia	≤ 20 21.6% 21–25 34.3% 26–30 28.6% ≥ 31 15.3%	Illiterate 31.0% Primary/read & write 39.0% Secondary school 18.3% Post-secondary school 11.0%	Housewife 42.7%] Farmer 23.7% Trader/Merchant 17.0% Government employee 10.0% Others 6.7%	Urban (54.7% Rural (45.3%	NR	Primigravida 13.7% Multigravida 41.3%	Paracetamol 72.7% Diclofenac 11% Antipain medicine 8.7% Ibuprofen 3% Tramadol 0.7% Amoxicillin 5.7% Cloxacillin 0.3% Metronidazole 0.3% Antibacterial medicines 0.3% Antacid c 3.3% Omeprazole 0.3% Metoclopramide 0.3% Hyoscine butylbromide 0.3% Magnesium sulphate 0.3% Anthelminthic medicines c 1% Ferrous sulphate 0.3% Multivitamin Tablets 0.3% Hydralazine 0.3% Name forgotten Medicines 1%	NR	NR	NR	Islam 66% Orthodox Christian 29.3% Protestant/Others 4.7%	Oromo 68.7% Amhara 9.0% Yem 8.7% Dawuro 5.0% Others 8.7%
Ake	2021	Nigeria	18–23 1 31.5% 24–29 37.0% 30–35 25.6% > 35 5.9%	No formal education 32.9% Primary 46.8% Secondary education 14.2% Higher education 6.1%	House wife 62.7% Farmer 6.1% Private 10.6% Government employee 6.1% Merchant 9.8% Other c 4.7%	Urban 46.0% Rural 54.0%	income in ETB < 500 44.9% 501–1000 18.4% 1001–1500 10.3% 1501–2000 8.9% > 2000 17.5%	NR	Paracetamol 6.1% Amoxicillin 2.5% Otherf 2.5%	Headache/fever/ pain 16.7% Gastritis(ant-acid) 4.5% Cough 3.6% Bacterial 1.4% Intestinal parasite 1.4%Othere 0.6%	Relative 10% Neighbor 10% Myself 6.1% Pharmacist 4.7% Husband 4.2% Other health professionals 3.1% HEWs 1.1% Radio 0.8% Otherh 0.3%	Lack of knowledge about medications danger 10.9% Prior experience 8.6% Save time 5.8% Save money 4.2% Medications easily available 5.0% Financial problem 2.5% Peer influence 1.1% No enough medications in the facility 1.1% Farness of health facility 1.1%	Protestant 77.4% Orthodox 51(14.2) Muslim 5.9% Catholic 2.2% Other a 0.3%	Gedeo 66.0% Oromo 13.7% Amhara8.4% Gurage 2.2% Other b 9.7%
Alonso-Castro	2018	Mexico	< 25 37.6% 25–34 43.9% > 35 18.5%	Elementary and middle school 38.3% High school 18% College-postgraduate 73.7%	NR	NR	NR	NR	Allopathy 26.6% Paracetamol 42.8% Bonadoxin 8.2% Omeprazole 5.2% Ranitidine 2.1% Dimethicone 2.1% Phenazopyridine 2.5% Antibiotic 3% Sennosides 3.6% Plantago 3.6% Antihistamine 4.1%	Nausea 27.9% Gastritis 14.7% Fatigue 11.7% Backache 8.1% Infection 12.2% Constipation 15.7% Migraine 22.6% Cold 20.8% Another symptom 20.1%	Relative/friend 59.4% Own initiative 24.4% Health professional 9.1% High risk 22.3% No risk 77.7%	Previous knowledge 32% Recommended 40.9% Lack of time/money 6.9% Fear of allopathy 6.1% Media 4.6%	NR	NR
Alsous	2020	Jordan	28.54 ± 8.37	Upper secondary school or less 37.7% Bachelor or diploma degree 59.0% Postgraduate degree 3.4%	Unemployed 37.7% Employed at non-medical field 59.0% Employed at medical field 3.4%	NR	NR	None 16.2% 1–3 pregnancies 45.2% 4 or more pregnancies 38.6%	Paracetamol 82.7% NSAIDs 2.8% Paracetamol and NSAIDs 2.8% Penicillin 6.9% Cephalosporin 6.5% Vitamins Multivitamins 30.9% Folic acid supplements 27.7%	Headache and joint pain 85.5% Common cold 11.8% Nausea and vomiting 9.0% Urinary tract infection 5.3% Others 8.3%	Knowledge from previous experience (61.8%) Advice from a physician (36.9%) Advice from a pharmacist (21.7%) Advice from a friend or a neighbor (5.8%) Social media (4.2%) Advice from a nurse (3.2%)	Previous experience with the disease 47.7%) The simplicity of the disease (36.6%) Medicinal products are easy to be obtained (21.2%) Time saving (14.7%) Limited access to the physician (4.4%) Long distance to reach a health-care faciliTY (1.8%) Others (1.6%)	NR	NR
Araujo	2013	Brazil	10 to 19:20.5% > 20 79.5%	< 8 years:26.9% > 8 years: 57%	NR	Metropolitan region 56.4%. Countryside 43.6%	< minimum wage 39.5%. > 1 + minimum wage 60.3%	1 to children 76.9% > 3 children 23.1%	Antianemic preparation 96.2% Vitamins 21.85 analgesics 57.7 corticosteroids 2.6% antibacterials 28.2%, gynecological antiinfectives and antiseptics. 25.6% antihistaminique 21.8% antiepelitic 1.3%psycholeptics 2.6%	NR	NR	NR	NR	Black/brown/indigenous 75.6% caucasian/Asian/Other 24.4%
Atmadani	2020	Indonesia	16–27 54.4% 28–45 45.6%	Middle school or lower 29.7% High school or higher 70.3%	Student 4.2% Homemaker 72.1% Employed 23.7%	Urban 73.0% Rural 27.0%	< 1.5 Million Rupiah 47.7% 1.5 Million Rupiah or more 52.3%	0 44.4% 1 or more children 55.6%	NR	Antiemetic medicines (33%), cold and flu remedies (29%), anti-fever medication (15%), pain killers (13%), and others (10%)	NR	NR	NR	NR
Befekadu	2014	Ethiopia	15–19 11(3.6%) 20–24 126 (41.6%) 25–29 115 (38.0) 30–34 36(11.9%) 35–39 15(5%)	Illiterate (6.3%) 1–8 (24.8%) 9–12 (37%) Higher Education (32%)	Housewife (24.4%) Merchant (23.8%) Employed (38.6%) Student (2%) Farmer (7.3%) Self employee (4%)	Urban 257 (84.8%) Rural 46 ( 15.2%)	Below 500 BIRR (19.8%) 501–1000 (29.7%) 1001–1500 (17.2%) 1501–2000 (16.2%) Above 2000 (17.2%)	No (46.5%) 1–3(49.2%) > 3 (4.3%)	Iron7 (11.5%) Paracetamol 21 (34.4%) Aspirin 8 (13.1%) CAF 5 (8.2%) TTC 1 (1.6%) Paracetamol and CAF 2 (3.3%) Paracetamol and TTC 2 (3.3%) ASA and CAF 1 (1.6%) Amoxicillin 4 (6.6%) cough syrup 6 (9.8%) Salbutamol tablet 4 (6.6%)	Cough 8 (13.1%) Typhoid 9 (14.8%) Headache 29 (47.5%) Common cold 1 (1.6%) Diarrhea 2 (3.3%) Anemia 8 (13.1%) Asthma 4 (6.6%)	Private drug retail outlets 44 (72.1%) Neighbors/Friend 3 (4.9%) Private drug retail outlets and shops 5 (8.2%) Shops and neighbors/friend 4 (6.6%) Private drug retail outlets and neighbors/friend 5 (8.2%)	Time Saving 20 (32.8%) Easily available 28 (45.9%) Better knowledge about the disease and the treatment 4 (6.6%) Time Saving and Easily av ailable 5 (8.2%) Time Saving and Better knowledge about the disease and the treatment 2 (3.3%) Easily available and Better knowledge about the disease and the treatment 2 (3.3%)	Muslim (47.5%) Orthodox (32.7%) Protestant(17.8%) Catholic (2%)	Oromo (54.8%) Amhara (18.2%) Gurage (7.3%) Welamo (5.6%) Dawro (5.3%) Keffa(5%) Others (3.96%)
Beza	2018	Ethiopia	18–24 (31.4% 25–29 (40.7% 30–34 (17.8% > 34 10%	No formal education (9.9)% Primary school (grades 1–8) 127.2% Secondary school (grades 9–12) 32.3% Certificate or diploma (21.6% First degree and above 9.1%	Housewife (37.1% Government employee 20.6% Private employee 27.6% Merchant 8.9% Housemaid 5.8%	NR	< 3000 ETB (30.6%)3000–5999 ETB (44.2% > 6000 ETB (25.1%	Primigravida (1) (41.2%) Multigravida (> 1) (58.8%)	Paracetamol 49.1% Amoxicillin 23.2% Tinidazole 4.5% Metronidazole 3.6% Ibuprofen 5.4% Albendazole 5.4% Panadol 6.3% Diclofenac 4.5% Mebendazole 1.8% Augmentin (amoxicillin + clavulanate) 0.9% Azithromycin 0.9% Tetracycline 2.7% Magnesium trisilicate 1.8% Ciprofloxacin 3.6% Aspirin 2.7% Bactrim (sulfamethoxazole + trimethoprim) 1.8% Ampicillin 1.8% Depo-provera (medroxyprogesterone) 0.9% Unknown medicines 2.7%	NR	Sharing with family, friends or neighbors 9.8% Pharmacy or drug store 76.8% Health facilities 0.9% Left-over medicines 21%. My SelF (64.3%) My husband, family or neighbors (29.5%) Pharmacist or druggist (7.14%) Nurse (2.75)	Time saving 26.8% Obtaining medicines easily 71.4% Disease not serious 54.5% Self-medication is cheaper 17.9% Previous medication experience 20.5% Being embarrassed to tell about the disease 1.8% High cost of visiting doctors and health service 5 4.5% Poor health service provision 0.9% Poor ethics of health professionals 0.9% Long waiting time for health services 19.6%	NR	NR
BiBintsene	2018	republic of Congo	67 + 6,3	Have school Attended 97,2% Not school attended 2,8%	Yes 68,8% No 31.3%	NR	NR	Multiparous 42,4% primiparous 57,65	Analgesics 94,4% Paracetamol 70,1% Diclofenac 17,4% Ibuprofen 6,9% Anti-malaria 7,6% Quinine 6,3% Arthemeter/ Lumefantrine 1,4% Antibiotic 13,2% Chloramphenicol 6,3% Doxycycline 1,4% Amoxicillin 2,8% Flucloxacillin 1,4% Cotrimoxazole 1,4%	Fever 27%, Headache 72%	market (55.56% %) and pharmacies 30.88%, family sotorage box (8%) and other sources (7%)	a strong belief in the safety of the drug (48.6%) and the lack of access to a health centre (47.9%)	NR	NR
Bobga	2021	Cameroon	15—20 23% 21—25 29.8% 26—30 19.2% 31—35 11.61% 36—40 9.6% ≥ 40 6.8%	Primary 32.7% Secondary University 48.07%	Student 17.4% Health personnel’s 8.4% Teacher 7.2% Farmer 28% Trader 13.6% Civil servant 11.6% Others 13.8%	NR	NR	NR	Cant recall 11.57% Antibiotics 12.76. Analgesics 17.21% Antiemetics 28.48% Antimalarial 8.60% Antiacids 21.36%	Fever/headache 21.36% Cough/cold 14.24% Nausea/vomiting 17.21% Constipation 18.69% Gastritis 15.73% Cannot recall 12.76%	Past prescriptions 19.88% Hospital/Pharmacy 14.24% Family/freinds/neighbours 34.42% Medicine stores 31.45%	High cost of medication in hospital/pharmacy 22.25% Previous experience 29.09% Non severity of ailment 20.77% Did not have money for consultation 20.77% Long distance from home to health facility 7.12%	NR	NR
Bohio	2016	Pakistan	18–26 13.6% 27–3554.5% > 35 31.8%	Illiterate 43.3% Primary 36.2% Secondary / Higher 20.5%	Housewife 95.2% Working 4.8%	Urban 34.5% Rural 43% Semi—urban 22.5%	Low 57.3% Middle 41.6% Upper 1.1%	Primigravida 21.9% Multigravida 67.2% Grand multigravida 10.8%	Acetaminophen + Aspirin 43.6% Acetaminophen + ibuprofen 18% Multiple drugs 12% / Ibuprofen15% / Multivitamins/Iron 3.8% Acetaminophen + Antacids 2.3% Aspirin 1.5% / Antibiotics 1.5% / Castor oil 0.8% / Unknown drugs 0.8%	Headache 60.2%Multiple complains 19.5% Headache + Backache 10.5% Fever 2.3% Headache + heartburn 2.3% Weakness 2.3% Constipation 0.8% Cough 0.8% Common cold 0.8% Suicide attempt 0.8%	Yourself 73.7% Yourself and husband 13.5% Pharmacist / drug store 11.3% Neighbors / Family members 1.5%	Better Disease knowledge 11.3% Attending health care is costly 31.6%. Easily Available 27.6% / timw saving 28.6% / Suicide Attempt 0.8%	NR	NR
MaliheBotyar	2018	Iran	< 20 0% 1–30 53.4% 31–40 42.5%. 41–45 4.1%	Illiterate 1.4%. Primary school 12.3%. Middle school 19.2%. Hight school 49.3%	Homemaker 86.3% Practitioner 13.7%	City 93.2%. Village 6.8%	No income 87.7% Under one million toman 12,3% between one and two milion 0	NR	NR	NR	NR	Easy access 4.3% Disease being mild 22.9%. The hight cost of treatment 2.4% Neglect 2,9%. No information one the disease 9% the drug being avalble at home 3.3%. Good results from previous self medication 1%	NR	Persian 42.5%. Turkish 21.9%. Lor 9.6%. Arab 0%. Other 19.2%
Bulabula	2019	South Africa	29.9 ± 5.9	primary school 4.9% secondary school 92.9% university 2.2%	NR	rural 55.8% urban 44.2%	≤ 250 USD 47.8% > 250 USD 52.2%	NR	NR	NR	NR	NR	NR	NR
Cabut	2017	France	[30.2 3.9]	NR	Executive and intellectual professions 22.1% Intermediate professionsc 36.8% Employees 30.9% Othersd 2.9% No occupation 7.4%	5000 inhabitants 77.6% > 5000 inhabitants 22.4%	NR	NR	NR	Headache 79.6% Sore throat 16.3% Stomach ache, gastroesophageal reflux 36.7% Dental pain 4.1% Other pains 18.4% Nausea, vomiting 18.4% Diarrhoea, constipation 4.0% Fever 8.2% Cough 4.1% Infectious disorders 12.2% Insomnia, stress 4.13% Otherb 4.1%	NR	NR	NR	NR
Navaro	2018	Italy	< 20 10.9% 21_25 19.9% 26–30. 25.1% 31–35. 24.6% > 35 19.5%	No formal education 10.3% Midlle school 37.4% Highr school.40.8% College dergree or higher 11.5%	Employed 44.6%. Unemployed 55.4%	NR	NR	Nulliparous 40.2%. Parous 59.8%	The most commonly prescribed medications were progesterone (14.6%), amoxicillin (8.7%), levothyroxine (8.3%), fosfomycin (7.3%), acetylsalicylic acid (7.3%), methyldopa (5.7%), and paracetamol (5.3%). The most commonly classes of medications used for self-medication were for nervous system and for alimentary tract and metabolism. The most frequently medications self-used were paracetamol (69.7%) and aluminum hydroxide (10%)	obstetric disorders (28%), urinary tract infection symptoms and other infections (17.4%), digestive disorders (8.3%), hypo/hyperthyroidism (8.7%), fever/common cold symptoms (8.7%), and hypertension (7%)	81.3% had received information about medications use during pregnancy and physicians were the most common source (75.3%), followed by internet (46.9%), and pharmacists (14.7%), whereas, only half had received information on the risk for the fetus (52.3%) and the majority of them from Gynecologists (86.3%). Finally, 57.8% would welcome in learning more about medications use during pregnancy	physician’s advice were a not serious disease (47%), advice/information by pharmacists (29.7%), they knew that the medication is safe during pregnancy (14.6%), and emergency care (13.5%)	NR	Italien 80.1%. Other 19.9%
Ebrahimi	2017	Iran	26.33 ± 4.60	Illiterate 1.0% Elementary 5.7% Guidance 11.7% Diploma 43.0% Higher Education 38.5%	Housewife 16.7% Employed 83.3%	NR	NR	NR	NR	NR	NR	NR	NR	NR
Gbagbo	2020	Ghana	15–20 8.85% 21–25 17.6% 26–30 36.8% 31–35 16.9% 36–40 14.0% 41–50 4.4% 46þ 1.5%	Tertiary 22.8% Secondary 34.6% Basic 38.2% No education 4.4%	Student 14.8% Self-employment 62.5% Paid employment 17.6% Unemployed 5.1%	NR	NR	Primiparous 29.4% 1 22.1% 2 20.6% 3þ 27.9%	Antibiotics 34.3% Pain killers 29.9% Antacids 7.5% Herbal medicines 28.4%	Headache 55.2% Cold and flu 3.0% Lower abdominal pains 17.9% Vagina infection 3.0% Malaria 9.0% Body pains 11.9%	NR	Reduced cost of treatment 25.4% Simple disease condition 43.3% Previous experience 20.9% Long waiting time 10.4%	Christian 86.0% Muslim 11.8% Others 2.2%	NR
Grover	2019	USA	NR	NR	NR	NR	NR	NR	NR	NR	NR	NR	NR	NR
Haque	2016	Bangladesh	15–20 35.4% 21–25 30.4% 26–30 24.1% 31–35 7.6% 36–40 2.5% > 40 0	Illiterate 11.4% Primary 54.4% Secondary 27.8% Graduation 6.3%	Service Holder 7.6% Student 2.5% Housewife 89.9%	NR	< 10,000Tk 84.8% 10,000–20,000Tk + 13.9% > 20,000Tk 1,3%	NR	antacid (27.8%) NSAIDs (26.6%), iron (15.2%), vitamins and minerals (12.7%), ayurvedic preparations (5.1%) and antiemetic (5.1%). antibiotics (3.8%), anti-histamine (2.5%) and phenobarbitone (1.3%),	gastric acidity (32.9%), infection (24.1%), cold & fever (21.5%), pain (12.7%), vomiting (8.9%), disorders related to pregnancy (7.6%), dysentery, diarrhea & food poisoning (5.1%), dental carries & toothache (3.8%) and anemia (2.5%). The obsessive conditions that imposed the minimal measure of pregnant women to take the medications without prescriptions were hypertension, skin diseases, allergic disorder, UTI, gonorrhea and sore throat	NR	availability of drugs (42.0%), pre-experience (20.8%), emergency usage (11.2%), knowledge about drugs (10.0%), advice from traditional healers (8.0%), non-serious illness (5.1%) and lack of faith in doctor’s practice (2.9%)	NR	NR
Barhamji	2014	France	29,7 (± 5,3)	NR	Cadre, profession intellectuelle supérieure 12,5% Employé 42,3% Agriculteur, artisan, ouvrier,15.1% profession intermédiaire 30.1% 15,1 16,1 12,4 Sans profession 30,1	NR	NR	NR	NR	NR	NR	NR	NR	NR
Ibrahim,	2021	United ArabEmirates	< 20 (2%) 20–29 (31.3%) 30–39 (37.6%) 40–60 (29.1%)	Uneducated (3.0)% High school (22.3% Undergraduates 10.6% University graduates 64.1%	Housewives (63.8% Health-related careers (7.1%) Other types of employment (29.1%)	NR	NR	1 (18.7%) 2–3 (47.9%) > 3 (33.4%)	Paracetamol (acetaminophen) 38 (25.3%) Multivitamins 28 (18.7%) Folic acid 18 (12.0%)	NR	Gynaecologists (77.9%) Pharmacist (13.1%) General practitioner (8.5%) Media and Internet (0.5%)	NR	NR	Emiratis (27.2)% Other Arab nationality (67.3)% Non-Arab 24 (5.5)%
Jambo	2018	Ethiopia	18–23 34% 24–29. 41.4%. 30–35 19.3% > 35. 5.3%	Illiterate 16.4%. Primary 1–8 38.9%. Secndary 32%. College universitystudents. 2.9%. Diploma/degree 9.8%	Goverment employee 10.2%. House wife 29.1%. Farmer 14.3%. Students 3.3%	Urban 80.7%. Rural 19.3%	< 13808poor 13.5%. 1381–6900 low. 84%. 6900–13800 middle 2.5%	No child 6.3%. one child 47.1%. Two children 27.6%. More than two children 19%	Paracetamol 33.8%. Cough Syrup 23.9%. Don't remember 22.5%. Amoxicillin 18.3%. Metronidazole 1.4%	Common cold 42.3%. Headache 36.6%. Nausea/vomiting 14.1%. Others 7%	Themselves 21.1%. Husband 1.4%. Freind 28,2%. Neighbour 18.3%. Pharmacist7druggist 23.9%. Other Health professional 7%	NR	NR	NR
Kabamba	2014	Dempocartic republic of Congo	16–20: 22 15,1% 21–30: 84 57,9% 31–40: 39 26,8%	Primary 15 10,3% Secondary 97 66,8% High education 33 22,7%	Households 17 11.7% Primary Students 3 2% Students 5 3.4% Informal sector 108 74.4% Female employees 12 8.2%	NR	NR	NR	Anaflam® (Ibuprofen + Paracetamol) 5% Aspirin® (Acetylsalicylic acid) 49% Dolarene® (Carisoprodol + Diclofenac) 7% Ibucap® (Ibuprofen + Paracetamol + Caffeine) 19% Ibuprofen 23%	NR	NR	NR	NR	NR
Karami Matin	2016	Iran	19–25 34.3% 26–36 28.6%	Under diploma 21.7% Diploma 25.5% Academic education 45.2%	Employed 40.5%. Housewife 27.3%	NR	NR	NR	POMs 9.2%. Pain medication 8.4%. Antibiotics 7%. Vitamins 3.3.%	NR	NR	NR	NR	NR
Leke	2018	Cameroon	13–17 58.5% 18–25 75.0% 26–35 72.5% 36–45 76.9%	Never went to school 68.4% Primarya 60.0% Secondary 74.5% High School 78.3% University/Professionala 88.5%	NR	Urban 71.5% Rural 75.3%	NR	0 73.1% 1 76.7% 2 76.6% ≥ 3 64.0%	Analgesics 48.8% Antianaemias 38.6%. Antimalarials 33.6%. Antibacterials 20.8%. Mineral supplements 14.5%. Vitamins. 8.8%. Other Medications 6.3%. Anifungal for dermatological use 5.7%. Antiinflamatory and antirheumatic. 4,9%. antihelminthics 4.4%. drugs for gastrotestinaldisordres 4.3%. Vaccines 3%. drugs for acid related disrodrs 2.6%. antiemetics and antinauseants 1.9%. antiretroviral 0.4%	n	NR	NR	NR	NR
Liao	2015	China	20–25 6.2% 26–34 80.8% 35–42 13%	High school and below 23.0% College and above 77.0%	NR	NR	≤ 800USD 41.2% > 800USD 58.8%	Parous 18.7% Nulliparous 81.3%	NR	NR	NR	NR	NR	NR
Lutz	2015	Brazil	≤ 19 32.4% 20–29 27.9% 30–47 25.8%	0–4 31.2% 5–8 32.1% 9–11 29.6% 12 or more 21.3%	NR	NR	≤ 1 30.1% 11–3 30.7% 3.1–6 27% 6.1–10 17.4% > 10 17.1%	1. 25.2% 2. 27% 3. 34.1% 4 or more. 37%	A—Alimentary tract and metabolism 13.7%. N—Nervous system 23.7% J—Anti-infectives for systemic use. 1.1%. C—Cardiovascular system2%. G—Genitourinary system and sex hormones. 4.5%. R—Respiratory system. 14.2%. H—Systemic hormonal preparations. 0.3%. M—Musculo-skeletal system 35.4%. B—Blood and blood forming organs 11.5%. D—Dermatologicals 11.1%. P—Antiparasitic products, insecticides 27.8% S—Sensory organs 16.7% L—Antineoplastic and immunomodulating agents 0	NR	NR	NR	NR	White 27.6% Black 26.2% Mixed/other 30.1%
Marwa	2018	Tanzania	18–27 47.06% 28–37 45.32% 38–47 45.45% > 47 0 0.00%	No formal education 65.00% Incomplete Primary school 65.79% Primary School 52.46% Secondary School 43.08% College or University Level 22.58%	Employed 28.57% Business 48.08% Unemployed 48.78% House wife 52.23%	NR	NR	1 45.83% 2 43.97% 3 46.59% 4 50.00% 5 50.00% 6 57.14% 7 100.0%	Antimalarial (24.42%) Antibiotics (9.58%) Antiemetic (34.30%). Analgesics (19.19%) Antiasthma (1.74%) Antiepileptic (1.16%) Antihypertensive (1.16%) Cough & Cold Remedies (5.23%) Heartburn (1.74%). Antihelmintics 1.16%)	Malaria 32.56% Urinary Tract Infection 9.3% Morning Sickness 25.55% Heartburn 2.34% Headache 19.19% Asthma 1.74% Epilepsy 1.16% Hypertension 1.6% Cough & Cold 5.25% Diarrhoea 1.58% Helminth’s 1.58% None 0 (0%) Fungal Infection 0.58%	NR	NR	NR	NR
Mbarambara	2016	Dempocartic republic of Congo	15–19 28.0% 20–24 38.6% 25–29 19.3% 30–35 11.0% > 35 3%	Illiterate 7.65% Primary school 23.9% Secondary school 61.8% University. 6.6%	House-wife 57.4% Employed 19.5% Student 20.5% Merchant 33.6% Self employed 5.0%		NR	NR	Paracetamol 40.8% Aspirin 7.6% Amoxicillin 17.1% Cough syrup 8.0% Vitamins 5.3% Papaverin 13.4% Irons 2.5% Vermox 1.3% Other drugs (antibiotics…) 1.8% Antacids 2.2%	Malaria 23.2% Typhoid fever 17.7% Tract urinary infection 43.9% Common cold and cough 30.5% Gastrointestinal disorders 7.1% Other diseases (anemia, asthma, headache, …) 27.9%	Pharmacist/druggist 73.0% Yourself 9.2% Free sale at the market 13.0% Neighbors/friends 4.8%	Disease not serious 28.3% Prior experience about the drug 25.4% Economical cost 14.1% Time saving 7.8% Easy access to the drugs without prescription 18.1% Others 6.2%	NR	NR
Mehmood	2018	Pakistane	18 – 25 13.7% 26 – 35 54.4% > 35 31.9%	Illiterate 43.3% Primary 36.2% Secondary / Higher 20.5%	Housewife 95.2% Working Women 04.8%	Semi-Ruler 34.5% Ruler 43.0% Urban 22.5%	Low 57.3% Middle 41.6% Upper 01.1%	Primigravida 22.0% Multigravida 67.2% Grand multigravida 10.8%	Acetaminophen + Aspirin 43.6% Acetaminophen + ibuprofen 18% Multiple drugs 12% Ibuprofen 15% Multivitamins/Iron 3.8% Acetaminophen + Antacids 2.3% Aspirin 1.5% Antibiotics 1.5% Castor oil 0.8% Unknown drugs 0.8%	Headache 60.2% Multiple complains 19.5% Headache + Backache 10.5% Fever 2.3% Headache + heartburn 2.3% Weakness 2.3% Constipation 0.8% Cough 0.8% Common cold 0.8% Suicide attempt 0.8%	By self 73.7% Husband and by self 13.5% Pharmacist / drug store 11.3% Neighbors / Family members 01.5%	Easily Available 27.6% Attending Health care is Costly 31.6% Better Disease Knowledge 11.3% Suicide Attempt 0.80% Time Saving 28.6%	NR	NR
Niriayo	2021	Ethiopia	< 18 2%. 19–25. 46%. 26–30. 33%. 31–35. 13.6% > 35. 6.4%	No formal education (14)% Primary school 16.4% Secondary 43.2%% higher education 26%	Civil servant. 23.6%. Merchant 20%. Housewife 46.4%. Others 10%	Urban 73.2% Rural 26.8%	< 5000 50.4%. > = 5000 49.6%	Primigravida 43% Multigravida 66%	NR	Morning sickness 39.2%. Headache 34.3%. Upper respiratory tract infections. 29.4%. Urinary tract infections 19.6%. Cough and cold 15.7%. Diarrhea. 13.7%. Allergic rhinits 7.8%	Pharmacies/drug stores 24%. Leftover medicine. 12.8%. Sharing with family,freinds or neighbors. 4%	easily accessing medicines. 25.5%. Diseas not serious. 21.6%. Timesaving 19.6%. Poor health service provision 14.7%. Cost saving. 12.7%. Ack of trust on prescribers. 5,9%	NR	NR
Odalovic	2012	Serbia	< 30 (68.8%) > 30 (31.2%)	< 12 (68.1%) > 12 (31.9%)	NR	Central (34.4%) Northern (30.2%) Western (35.4%)	< 300 (73.3%) > 300 (26.7%)	< 1 (54.1%) > 1 (45.9%)	NR	NR	NR	NR	NR	NR
Ojo	2020	Nigeria	15–20. 3.2% 21–26. 29.7% 27–32. 43.4% 33 and above 23.6%	Primary. 8.2% Secondary. 42.3% Tertiary. 49.6%	NR	NR	NR	NR	NR	NR	NR	Drugs prescribed in hospital are expensive 53.9 5 The illness is not serious/minor 65.0% There is no time to go to the health facility 39.9% Going to hospital for treatment waste time 31.5% The cost of the health practitioner treatment is high 45.2% Availability of an old prescription 47.8%	Christian 85.7%Muslim. 12.8% Traditional. 0.6%. Others. 0.9%	Yoruba 80.8% Igbo. 10.2% Hausa. 2.6% Others. 6.4%
Pakseresht	2020	Iran	16–25. 24.4% 26–35. 59.7% 36–45 15.4% > 46. 0.5%	Reading & Writing 37.5% Diploma & Higher 62.5%	Employee 6.7% Self-business 5.8% Housewives 87.5%	Rent 43% Owner 51% With family 6%	< 300 $. 27.6% 300–600$. 67.9% > 600$. 4.5%	0 17.6% 1–2 77.3% 3–4 5.1%	Analgesics 50.94%. Antihistaminics 9.43% cold syrup5.56%. Antinausea and vometting medecine 3.275%. Calcuim supplement 1,89%	headache (34%), common cold (22.6%), and digestive diseases (22.6%)	Physician. 1% Family. 0.2% Neighbor. 0.2% Radio-TV. 2.2% Book- pamphlet 4.1% Internet 20.6% More than 1 item	Fear of medication side effects 83.4% Uncertainty about drug effectiveness. 12.4% Influenced by the family. 4.2%	NR	NR
Pereira	2021	Brazil	< 30 43.9% 30–39 48.6% > 40 87.4%	Elementary school 10.2% High school College and university 89.7%	Employed 58.8% Unemployed 8.4% House wife 32.7%	NR	0–2 72.1%. 3–5. 23%. > 5. 4.8%	1. 18.6%. > 2 81.3%	acetaminophen 35.1% metamizole 28 Metamizole association 17.7% ibuprofen 9.3%. Scopolamine 5.6%. Others 19.1%. Naphazoline 4.6%. Dimenhydrinate 3.7%. Omeprazole 2.8% guaco syrup 2.8%. Mutivitaine 1.8% passionflower 1.8% others 7.2%	hypertensive syndrome during pregnancy 29.2%. Diabetes mellitus during pregnancy 28.2%. Thyroid disease 12.7%. Collagen disease and antiphospholipid syndrome 8.3%. Bad obstetric history 6.7%. Nervous system problems 6.3%. Uterine anomaly and preterm labor 6% infection 6%. pulmonry disease 4.7%. Mental health 4.7%. obstetrics complications 3.7%. diagnosis under investigation 6% others 20.5%	Neighbor or friend 11.2%. Lefover from pervious treatement 10.2%. Family memeber 8.4%. Pharmacy 8.4%. Internet 5.6%. Others 4.5%	Considred simple health problem 62.6% Practicality 50.4% Lack of access to health system 23.4% others 12.1%	NR	White 47.6%. No white 52.3%
Pisa	2015	Italy	< 25 6.6% 25–29 13.8% 30–34 38.4% 35–39 32.7% 40 + 8.5%	< High school 15.75 High school 45.9% University 38%	Employed on maternity leave 73.8% Currently employed 7.2% Housewife 8.5% Unemployed 6.6%	Italy 91.5%. Other 7.9%	NR	NR	NR	NR	NR	NR	NR	NR
Rafiee	2018	Iran	< 25 years 20.5% 25–30 years 30.4% > 30 years 27.4%	Diploma 39% Academic 38.2%	Housewife 25.8% Employed 22.2%	Urban 30.7% Rural 13.3%	< $150 21.7% $150–300 29.1% $300–600 37% > $600 33.3%	NR	NR	NR	NR	Belief in the treatment of disease by medications alone 77(74.8) Safety of medications 77(74.8) The availability of medications at home 73(70.9) Inappropriate knowledge of the effects of drugs 68(66) Desirable results of the previous self-medication by the mother 64(62.1) Previous experience of similar disease 62(60.2) Ignoring the importance of the disease by the mother 62(60.2) Easy drug delivery from drug store 51(49.5) Expensive visit fee of doctors 27(26.2) Insufficient time 23(22.3) Lack of access to doctor 21(20.4) No insurance coverage 16(15.5) Insist of friends 14(13.6) Uncertainty about doctor’s practice 10(9.7)	NR	Arab 21.3% Bakhtiari 41.4% Fars 50% Others 16.7%
Raheel	2017	Saudi Arabia	< 20 (2.5%) 20–30 (67%) 31–41 (25.5%) > 40 (5%)	Elementary (3%) Intermediate (5%) High school (35%) Bachelor (50%) Higher education (7%)	House-wife (53%) Student (14%) Health-related employee (4%) Teacher (16%) Others (13%)	Saudi (91%) Non-Saudi (9%)	< 3,000 (5%) 3,000–4,999 (20%) 5,000–9,999 (47%) 10,000–20,000 (24%) > 20,000 (4%)	1 (43%) 2 (18%) 3 (15%) 4 (8%) > 4 (16%)	Antibiotic 3%. Paracetamol 22%. Aspirin 3%. Ibuprofen 3%. Antihistaminics 3%. Antiacid 6%	NR	pharmacists (53%), medication pamphlets (28%), media (27%), family member (11%), and friends (4%)	NR	NR	NR
Sah	2020	Nepal	18–23 31.6%, 24–29 41.3% and 30–35 42 (18.7%) > 35 19 (8.4%)	NR	(63.6%) house wives (36.4%) were employed	NR	NR	NR	Paracetamol 28% Cough 18%. Analgesics 10%. Antiemetics 15% Antibiotics 9%. Others 13%	headaches (31.2%), treat fever (11.8%), cough (22.6%), vomiting (14%), urinary tract infections (11.8%) and others (8.6%)	Friends 28% Health professionals 23.6% Neighour 12.9% Social netework 11.8% Others 15.1% Husband 8.6%	Disease were not serious 46.2% High cost to visit doctor 29% It is time saving 19.3% Drugs are easily available 5.4%	NR	NR
Sema	2020	Ethiopia	18–27 48.2% 28–37 49.8% > 38 2%	Illiterate 5.8% Primary school [1–8] 14.3% Secondary school [9–11] 37.8% College/university student 1.8% Diploma/degree 40.5%	Governmental employed 31.3% Self-employee 26.5% Housewife 31.5% Farmer 5.8% Student 2.3% Unemployed 11%	Urban 84.35 Rural 15.8%	< 3000 39.8% 3000–6000 33.8% > 6000 26.5%	No child 31.3% One child 39.8% Two children 21.0% More than two children 8.2%	Paracetamol 58.0% Amoxicillin 16.0% Cough syrup 4.0% Hyoscine 4.0% Metronidazole 8.0% I do not know the name of the drug 10.0%	Headache 60.0% Nausea/vomiting 6.0% Typhoid 8.0% Common cold 8.0% Cough 16.0% Unspecified 2.0%	From friends 8.0% Internet 6.0% Pharmacist/druggist 74.0% Other health professional 12%	Health problem 3.5% Low economic level 0.3% Unwanted pregnancy 1.3% Unspecified 1.5%	Orthodox 76.3% Muslim 20.0% Protestant 3.5% Jehovah witness 0.3%	NR
Sina	2021	Ghana	18—24. 19.6% 25—34. 68.9% 35 – 45. 11.5%	No formal education 45.5% Primary. 15% JHS. 12.8% Secondary + 26.7%	Students 6% Self-employed/ private business 45.5% Government 12.3% Unemployed 36.2%	NR	NR	None 30.2% 1. 29.2% 2. 25.9% 3 + . 14.7%	(Paracetamol) 76.1% NSAIDS (Ibuprofen, Diclofenac) 12.5% Tramadol 32.4% Antibiotics (e.g. amoxicilin, septrin, flagyl, etc.) 22.1% Dewormers 2.6% Antimalarial 16.2% Herbal medicine (e.g., Achyranthes aspera, sphaeralcea coccinea, etc.) 10.3% Anti-dyspeptics (e.g., antacids, omeprazole, etc.) 9.9% Cough syrup 7.7% Anti-emetics (e.g., promethazine, etc.) 6.6% Vitamins (vitaprime, ultimate omega, B-G glutamine plus and ultra-vitamin B complex) 2.6% Diazepam (valium, diastat and diastat AcuDial) 3 1.1	headache (34.2%), [upper] back pain (33.1%), waist pain [low back pain without sciatica] (32.7%), lower abdominal pain (20.6%) and malaria (16.2%)		Perceived illness as minor 60.8% Prior experience with self-medication 39.5% Availability of health facilities 56.7% Far distance to health facilities.50.4% Availability of enough skilled health personnel 70.3% Waiting time at health facility 24% Number of times told there is no medicine at the13.6% Difficulty getting transportation to the health 53.1% Cost of transportation to the health facility 33.8% Availability of chemical shops and pharmacies 90.5% Closeness of chemical shops 23.7% Cost of medicine bought from the chemical shop 39.2% Presence of cultural norms and beliefs that promote 31.1% Potency of traditional medicine over orthodox medicine 17.5%	NR	NR
Tefera	2020	Ethiopia	NR	Illiterate 38.3% Primary school 29.2% Secondary school [9–11] 29.7% College/university student 2.9%	Student 4.9%. Governmenet employee 14.3%. Merchant 16.4%. Farmer 4.7%. House wife 53.4%. Selfemployed 6.3%	Urban 72.7% Rural 27.3%	< 500 6.3%. 500–1499 11.7%. 1500–2499 50.6%. 2500 and above 31.3%	NR	Amoxicilin 1%. Tracycline 5%. Paracetamol 1%. Misoprotosl 0.5%. Wafarin 0.5%	NR	Pharmacists 41.9%. Nurse and Midwives 22%. General Practioner 21.3%. Gynocllogist 13.8%	NR	Orthodox 49.7% Muslim 34.1% Protestant. 8.1% Others 0.5%	Amhara 61.9%. Tigre 36.2%. Qimant 4.7%. Others 1.8%
Tuha A	2020	Ethiopia	15–19 26% 20–24 10.8% 25–29 31.8% 30–34 16.6% 35–39 10.8%. 40–49 4%	Illiterate 19.7% Primary school [1–8] 17% Secondary school [9–11] 8.1% College/university student 38.6% Diploma/degree 16.6%	NR	Urban 70% Rural 30%	1000–1400 18.4%. 1500–1900 2.2%. 2000–2900 40.8%. 3000–3400 15.7%	1–5 96%. > 5 4%	Paracetamol 31.7% Amoxicillin 20% aspirin 6.7%. Cough syrup 28.3%. Hyoscine 13.3%	Headache 30.0% Nausea/vomiting 15% Common cold 28.0% Cough 20.0% urinry tract inection 7.0%	Yourself 23% husband 7% Pharmacist. 43%. /drug store Neighbors / Family members 13%. Internet 13%	Time saving 19.8%. Easily available 14.4%. Poor knowledge about disease and drug 19.8%. Prior experience to the drug 46%	NR	NR
Verstappen	2013	Pays bas	18–35 12.2% > 35 15.1%	Low 8.0% Middle 13.3% High. 12.8%	NR	NR	NR	0 15.7% 1 10.4%	Dermatics, Homeopathy, Comforting medication ± 12.8% Analgesics‡ 27.3% Cold & flu medication 11.8% Prenatal vitamins and other vitamins† 26.7% Gastro-intestinal tract medication 21.45%	NR	NR	NR	NR	Western 12.3% Non-western 23.1%
Wakjira	2019	Ethiopia	21–30 50.3% 31–40 35.4% > 41 14.4%	Illiterates 17.4% Primary 12.8% Secondary 12.8% College/university 19.5% Diploma/degree 37.4%	Government employee 27.7% Self-employee 32.3% Farmer 6.2% House wife 15.9% Student 17%	Urban 81.5% Rural 18.5%	< 1000 5.6% 1001–2000 7.2% 2001–3000 17.9% > 3000 69.2%	No 21.0% One 19.0% Two 26.2% Three 21.5% ≤ Four 12.3%	Paracetamol 45% Amoxicillin 19% aspirin 7%. Other 27%	Common cold 21%. Headache 33%. Nausea/vomiting 21%. Diarrehoea 27%	Your self 2.7% Your neighbor 40.5% Pharmacist/druggist 57.1%	Time saving 2.4% Better knowledge about the disease and the treatment 38.1% Had prior experience to the drug 45.2% Easy available 14.3%	NR	NR
Yusuff	2011	Nigeria	NR	Primary 34.5% Secondary 42% University 16.1% None 7.4%	Self-employed (traders and artisans 61.5% Unemployed 25.1% Civil servants 13.4%	NR	NR	NR	Paracetamol 556 (34.3) Hematinics ? vitamins 548 (33.8) Local herbs 190 (11.7) Piroxicam 91 (5.6) Cough medicines 88 (5.4) Dipyrone 44 (2.7) Ampicilin 31 (1.9) Chloramphenicol 26 (1.6) Prednisolone 23 (1.4) Diazepam 13 (0.8) Calcium supplements 13 (0.8)	Body pains/fever (30.1%) Joint pain (14.5%) Cough (10.2%) General weakness (9.2%) Indigestion (8.5%) Headache (7.8%) Sleeplessness (7.6%) Nausea (7.2%) Heart burn (2.5%) Body swelling (2.4%)	Mother-in-law ? relatives (41.3%) Patent medicine vendor (20.2)% Pharmacist (12.8%) Nurse (10.1%) Neighbour (8.3%) Traditional healer (7.3%)	Unrestricted availability of medicines (40% Long distance to public health facilities 29.6% Financial difficulty 18.5% Perceived poor service delivery at facilities 11.9%	NR	NR
Zaki	2013	SaudiArabia	20–30 (34.2%) 30–40 (44.7%) 40–50 (21.1%)	Illiterate (7.9%) Primary (6.6%) Secondary (18.4%) University (65.8%) Postgraduate (1.3%)	House-wife (81.6%) Student (1.3%) Health-related career employee (7.9%) Other employee (9.2%)	Rural 750 Urban 10	NR	First-time pregnancy (15.8%) 1–3 previous children (28.9%) More than 3 previous children (55.3%)	Paracetamol (13.2%) Antacid (1.3%) NSAIDs (1.3%) Antibiotics (2.6%) Antihistaminics (1.3%) Drugs for nausea and vomiting (2.6%) Vitamins (13.2%) Herbs (4.6%) No medication (59.9%)	NR	gynecologist (58.1%) then general practitioner (GP; 13%) and pharmacist (11%). The media,5% family and friends as well as the internet collectively contribute by 10%	NR	NR	Saudi (71.1%) Egyptian (7.9%) Sudanese (3.9%) Jordanian (3.9%) Syrian (6.6%) Indian (1.3%) Pakistanis (2.6%) Others (2.6%)
Zewdie	2018	Ethiopia	15–24 29.1% 25–34 63.8% 35 and above 7.1%	Unable to read and write 7.7% Read and write 10.8% Primar 31.9% Secondary and above 49.5%	Housewives 49.5% GOV Worker 20.7% Merchant 18.3% Student 2.5% Self-employee 8.7% Othersb 0.3%	NR	NR	One 39.6% Two 35.9% Three and abovE 24.5%	NR	Vomiting 25% Back pain 17.3% Headache 11.5% Heart burn 21.2% Constipation 11.5% Cough 5.7% Othersa 7.7%	NR	NR	Orthodox 40.6% Muslim 47.1% Protestant 10.8% Catholic 1.2% Othersa 0.3%	Oromo 65.0% Amhara 23.5% Somali 2.5% Gurage 2.8% Otherc 6.2%
Andy Emmanuel	2014	Nigeria	15–20. 16.6%. 21–25. 37.5%. 26–30. 18.3%. 31–35. 15%. 26–40. 9.16%. > 40. 3.33%	tertiary 41.6% Secondary 24.2% primary 23.3% 38.2% other 10.8%	Goverment employee 20.8%. Employee by private busniess 23.3% self employee 25%. Students 12.5%. Unemployee 18.3%	NR	NR	NR	Analgesics 24.1%. Antimalarials 23.4%. Vitamins. 17.3%. Antacid 14.5%. Antibiotics. 16.5%. Heral remides 4.1%	Headache7fever 33.3% malaria 31.2% Common cold Cough 13.8% urinry tract inection 6.6. %. Gastrointestinal disorders 10%	NR	dOCTORS ARE SCARE AND EXPENSIVE TO DEE 18.3%. Prior experience about the drug. 22.5%. Illnessess are minor. 35%. No esponse. 13.3%. It's dangerous. 4.1%. It's good 6.6%	NR	NR
M. Sarani, S. Saravani	2017	Iran								Respiratory diseases 33.4%. Hypertension 26.9%. Diabetes 16.3%. Heart diseases 14.3%. Anemia 7.4%. Gatsrointestinal and psychiatric diseases 1.7%				
M. J. Twigg1	2016	UK	Mean age 30.5%	Less than high school 0.6% High School 27.9%. More than high school 52.1% Other 19.3%	NR	NR	NR	No previous children 48.0%	Piperazine derivatives e.g. cyclizine (R06AE) 16.7%. Other drugs for peptic ulcer disease and GORD e.g. alginic acid (Gaviscon) (A02BX). 84% Combinations and complexes of aluminium, calcium and (34.2% H2-receptor antagonists, ranitidine (A02BA) 3.4% Proton pump inhibitors, omeprazole (A02BC). Osmotically acting laxatives e.g. lactulose (A06AD) 66.4% Bulk-forming laxatives e.g. ispaghula (A06AC) 26 (20.2% Contact laxatives e.g. senna (A06AB) 22 (19.3%)	Nausea 78.6%. Constipation 55.3% Heart burn 74.3% Common cold 55.5% Urinary tract infections 17.1% Headache 62.8% Pain in neck or pelvic girdle 66.5% Sleeping problems 67.6%	NR	NR	NR	NR
D. Courrier	2015	france	< 20 1 (0,3%) 20—29 47,2% 30—39 49,1% ≥ 40 13,4%	Bac + 5 et plus 29,7% Bac + 3 et 4 22,6% Bac + 1 et 2 24,5% Lycée 20,9% Collège ou moins 2,3%	Professions intellectuelles supérieures (cadres) 11,8% Professions intermédiaires Professions de santé 17,9% Employées 12,4% Artisanes, ouvrières 38,1% Sans activité 15.6%	Urban 49.5% Rural 50.5%	NR	0 37,6% 1 38,2% 2 15% 3 6,9% 4 et plus 2,3%	Paracétamol Phloroglucinol Anacides Laxafs Compléments nutrionnels Eléments minéraux Protecteurs cutanés Vitamines Veinotoniques Andiarrhéiques Corcoïdes Anfongiques Carbocystéine An-inflammatoires non stéroïdiens Anbioques An-sécrétoires gastriques Ansepques locaux An-éméques Doxylamine Antipoux	Céphalées Infecons hivernales (toux, maux de gorge,…) Reflux gastro-oesophagiens Douleurs abdominales Conspaon Lombalgies Contracons utérines Fague Douleurs autres Nausées-vomissements Insomnies Douleurs ligamentaires Douleurs dentaires Fièvre Douleurs hémorroïdaires Anxiété Vergetures Diarrhées Prurit vaginal Herpès Troubles circulatoires Brûlures miconnelles Symptômes en lien avec antécédents Prurit cutané P	NR		NR	Franc¸aise (93,8%) Africaine (2,6%) Européenne hors France (2,35) Autre (1,3%)
Lupattelli	2014	Ausralia	≤ 20 62.3% 21–30 66.8% 31–40 67.4% ≥ 41 68.4%	Less than high school 69.7% High school 65.6% More than high school66.8% Others, unspecified 70%	Employed, but not as HCP 65.65% HCP (75.2%) Student 67.4% Housewife 68.9% Job seeker 60.6% Other than above 63.3%	NR	NR	No 62.6%. Yes 71.2%	OTC painkillers, Paracetamol (including combinations) Non-steroidal anti-inflammatory drugs ( Acetylsalicylic acid (including combinations) (N02BA) Metamizole (N02BB02) – OTC antacids, Antacids (aluminium, salts combinations, antiflatulents) Alginic acid complex/sucralfate/bismuth (A02BX) H2 receptor antagonists (A02BA) Antacids with calcium (A02AC)) Proton pump inhibitors (A02BC) OTC nasal sprays/drops, By drug group Sympathomimetic nasal decongestants Nasal corticosteroids (R01AD) Nasal immunostimulants (low-dose interferon) OTC laxatives, Osmotically acting laxatives (A06AD) Contact laxatives (A06AB) Enemas (A06AG) Softeners, emollients (A06AA) OTC antinauseants First generation antihistamines (R06A) Metoclopramide/domperidone/bromopride (A03FA)	NR	NR	NR	NR	Western Europe 67.6% Northern Europe 76.4%) Eastern Europe 57.5%) North America 64.2% South America 45.1% Australia (77.4%
		Italy												
		austria												
		Switzerland												
		france												
		Pays bas												
		UK												
		Norway												
		Sweden												
		Finland												
		Iceland												
		Russia												
		Poland												
		Croatia												
		Serbia												
		Slovenia												
		USA												
		Canada												
		South America												

### The source and quality of the information received

The choice of treatment for maternal illness was influenced partly by the source and quality of information on drugs received. Differences in over-the-counter drug use patterns and drug prescribing systems that differ from one country to another. For example, in some African countries, all medicines can be purchased without a prescription. However, Pharmacies or drug stores that were authorized to vend and supply drugs and relevant information left significant adverse implications on the overall health outcomes of users. This implies that women who engage in self-medication tend to be primarily guided by the origin of the information they receive and the quality of that information regarding medications. The majority of the studies pointed out various sources and the quality of information as key factors. First, some women opted for drug use during pregnancy based on their previous prescriptions and available medications within their family. Second, certain pregnant women received recommendations from their relatives (mothers), friends, and neighbors. Additionally, healthcare professionals (such as doctors and nurses) and online pharmacies also played a role in influencing women's decisions about medication use. Finally, other users were influenced by recommendations from sources such as the Internet, social media, newspapers, radio, and television.

## Discussion

The results of the present study showed that the global prevalence of self-medication among pregnant women in the world is 44.55% although the results show a high heterogeneity. This rate is different from the results of a systematic review and meta-analysis that investigated self-medication among pregnant women worldwide (32%) [10]. Our systematic review differs from the earlier study in several key aspects. Firstly, it provides a more comprehensive and up-to-date analysis of the existing literature, offering a more complete understanding of the subject. We've included a broader spectrum of studies [53], incorporating diverse geographical locations and socioeconomic settings. Moreover, our review delves deeper into the underlying factors and context surrounding self-medication, offering a more holistic perspective on the issue. In essence, this systematic review and metanalysis contributes a more nuanced and comprehensive examination of the relationship between income status, region, and self-medication, providing valuable insights for healthcare policymakers, practitioners, and researchers.

Despite the prevalence of self-medication being higher in pregnant women, due to the complications of pregnancy, Our results compared to the results of other studies showed that self-medication in pregnant women was relatively low in comparison with other groups in general. In Ethiopia, the prevalence of self-medication was found to be 44.0%. Geographical-based subgroup analysis revealed that the highest prevalence was observed in the capital of Ethiopia, Addis Ababa, 62.8%. Population-based analysis indicated that healthcare professionals and students were the main practitioners of self-medication. Besides, the prevalence of self-medication practice in pregnant women is approximately 22.9% [82]. A meta-analysis conducted in Iran among students showed a very high prevalence of 70.1% [83]. The results of the subgroup analysis in our study showed that the prevalence of self-medication varies by region, this finding is attributable to the difference in health-related socio-cultural knowledge, beliefs, attitudes, and behaviors among pregnant women from different geographical areas. The large difference in prevalence rates of self-medication in different regions of the world may be due to differences in over-the-counter drug use patterns and drug prescribing systems that differ from one country to another. In some African countries, all medicines can be purchased without a prescription [26]. As an example, the pooled prevalence of self-medication during pregnancy in Ghana was 65.4% (95% CI = 58.2%–72.6%; *I*2 = 88.32%; *p* < 0.001) [84]. Other reasons that may influence self-medication include the high cost of medical visits and limited health insurance coverage. Women with unplanned pregnancies are also more likely to self-medicate [69]. The low prevalence rate noted in Asian countries may be due to the cultural and social habits of using complementary and alternative medicine. The prevalence of the use of herbal medicines among pregnant women varied from 9.2% to 90.2% this was reported in a systematic review conducted by Eastern Mediterranean Regional Office [85]. Most pregnant women believe that herbal medicine is more effective than conventional medicine [85, 86]. Others think that are safe and secure for the mother and her fetus and have fewer side effects than conventional medicine during pregnancy [82, 87]. Stratifying by a score of the quality assessment of the studies, the differences in the prevalence of self-medication can be explained by the fact that high-quality studies have a low risk of bias and therefore the prevalence approaches the pooled prevalence (44.50%). However, the low-quality studies probably overestimated the prevalence because of the low recruited sample size. For the cohort studies, the prevalence was lower this can be explained by the fact that the research methodology of the cohort studies is very rigorous with the best evidence [88]. The results of the subgroup analysis in our study showed that the prevalence of self-medication varies by income status. The high prevalence rate noted in countries with high-income levels was 58.88% compared to the low-income countries the rate of prevalence was 37.07%. We can explain these results that lower-income individuals may have limited financial resources to access healthcare services, including doctor consultations and prescription medications [89, 90]. Health literacy refers to an individual's ability to understand and use healthcare information to make informed decisions. This knowledge and understanding about appropriate medication use could contribute to a higher prevalence of self-medication. Lower-income individuals may have limited access to health education and information, resulting in lower health literacy [89].

In recent years, several initiatives and interventions, such as improving the knowledge of pregnant women about the consequences of self-medication, as well as the provision of brochures and catalogs, have been planned and implemented, which could be very effective in combating this practice. In addition, the continuous training of health professionals on the prescription of drugs and the advice given when dispensing drugs to pregnant women could reduce the prevalence of self-medication. Despite the attempts made by nations to decrease the prevalence of self-medication among expectant women, this behavior continues to increase. Consequently, there is an immediate need to implement novel and more efficient preventive strategies.

### Strengths and limitations of the study

The strengths of our study are the large sample size, sample size analysis, and subgroup analysis. All these analyses reflect the methodological rigor of our systematic review and meta-analysis. On the other hand, the included articles provide large and profound information on various aspects of self-medication in pregnant women (prevalence, groups of drugs most used by pregnant women, groups of diseases most often treated by self-medication, and the most common reasons for self-medication) that can be used by health professionals to make decisions and organize effective interventions to prevent self-medication in pregnant women.

The limitation of this study was the fact that we included only articles in English and French, while we excluded some studies of Spanish and Farsi languages after exploiting their abstracts if they only reported the prevalence of self-medication. Another limitation of the present study was few databases were included and some more relevant ones were not searched. The limitations of the subgroup analysis are that gives both false positive and false negative results.

In addition, the quality of the included studies was different and the inclusion of some studies of poor quality may affect the final estimate.

## Conclusion

The results obtained from this study showed that the prevalence of self-medication among pregnant women is relatively high. This requires effective measures and interventions to reduce self-medication. We recommend that health professionals consider implementing programs on the risks of self-medication, and strengthening the control and monitoring of over-the-counter sales of drugs. Physicians and pharmacists should also be made more sensitive to prescribing the appropriate medication and avoiding the provision of medication without a prescription.

## Data Availability

Not applicable.
